# S100A4/TCF Complex Transcription Regulation Drives Epithelial-Mesenchymal Transition in Chronic Sinusitis Through Wnt/GSK-3β/β-Catenin Signaling

**DOI:** 10.3389/fimmu.2022.835888

**Published:** 2022-01-28

**Authors:** Ningyue Gong, Lei Shi, Xin Bing, Hui Li, Houyang Hu, Pan Zhang, Huiming Yang, Na Guo, Hongjie Du, Ming Xia, Chengcheng Liu

**Affiliations:** ^1^ Department of Otolaryngology, Shandong Provincial Hospital Affiliated to Shandong First Medical University, Jinan, China; ^2^ Department of Otolaryngology, Shandong Provincial Hospital, Cheeloo College of Medicine, Shandong University, Jinan, China; ^3^ Department of Biotechnology Research and Development, Qilu Pharmaceutical, Co.Ltd, Jinan, China; ^4^ Central Laboratory, Shandong Provincial Hospital Affiliated to Shandong First Medical University, Jinan, China

**Keywords:** CRS, epigenetic regulation, EMT, S100A4, inflammation

## Abstract

Epithelial-mesenchymal transition (EMT) is thought to be involved in the tissue remodeling and long-term inflammatory process of chronic sinusitis (CRS), but the driving mechanism is still unclear. Using high-resolution mass spectrometry, we performed a proteomic screen of CRS nasal mucosal tissue to identify differentially expressed proteins. Data are available *via* ProteomeXchange with identifier PXD030884. Specifically, we identified S100 calcium binding protein A4 (S100A4), an effective factor in inflammation-related diseases, and its downstream protein closely related to tissue fibrosis collagen type I alpha 1 chain (COL1A1), which suggested its involvement in nasal mucosal tissue remodeling. In addition, stimulation of human nasal epithelial cells (HNEpCs) with lipopolysaccharide (LPS) mimicked the inflammatory environment of CRS and showed that S100A4 is involved in regulating EMT and thus accelerating tissue remodeling in the nasal mucosa, both in terms of increased cell motility and overexpression of mesenchymal-type proteins. Additionally, we further investigated the regulation mechanism of S100A4 involved in EMT in CRS. Our research results show that in the inflammatory environment of CRS nasal mucosal epithelial cells, TCF-4 will target to bind to S100A4 and regulate its transcription. The transcription of S100A4 in turn affects the execution of the important signaling pathway in EMT, the Wnt/GSK-3β/β-catenin pathway, through the TCF-4/β-catenin complex. In conclusion, this study confirmed that the expression of S100A4 was significantly increased during the progressive EMT process of CRS mucosal epithelial cells, and revealed that the transcriptional regulation of S100A4 plays an important role in the occurrence and development of EMT. This finding will help us to better understand the pathogenesis behind the remodeling in CRS patients, and identify target molecules for the treatment of CRS.

## Introduction

The clinical prevalence of chronic rhinosinusitis (CRS) is high, affecting about 8% of the Chinese population (1), and more than 10% of adults in Europe and the USA are affected by the disease (2). The post-operative recurrence rate can be as high as about 20%, seriously affecting the quality of life of patients (3). As a result, in recent years, there has been an increase in the research of the pathogenesis of CRS. Currently, it is thought that the function of the epithelial barrier of patients with CRS is impaired under the influence of long-term inflammation, and tissue remodeling gradually occurs during the epithelial repair process (4, 5). Studies on chronic inflammatory diseases of the lower airways have shown that damage of the lung epithelial cell barrier leads to airway remodeling with inflammatory cell infiltration and altered bronchial structure (6). Since Grossman’s observation that rhinitis and asthma often coexist in the same patient, the idea of “one airway, one disease” co-morbidity has been proposed, as has impaired epithelial barrier function and tissue remodeling in chronic inflammatory diseases of the upper airways (7). The European Position Paper on Rhinosinusitis and Nasal Polyps 2020 (EPOS2020) guideline also classifies CRS comorbidities into separate chapters (8). Proteomics has been developed over the last few decades and is a very reliable method for screening differentially expressed proteins (DEPs). In a review of proteomic studies related to tissue remodeling in CRS, the nasal mucosa proteome showed increased protein expression of cellular components, such as cytoskeleton and adhesion junctions, in CRS patients compared to healthy subjects (9). The nasal mucus proteome showed dysfunction of immune pathways, decreased cell signal transduction, increased cell metabolism and related tissue remodeling pathways, and the mucosal immune and antioxidant pathways were significantly downregulated with the progression of tissue remodeling (10).

Epithelial mesenchymal transition (EMT), which is the morphological transformation of the epithelial cell phenotype to a fibroblastic or mesenchymal cell phenotype, may explain epithelial dysfunction and tissue remodeling. Under normal physiological conditions, the main structures that maintain the epithelial barrier are the tight junctions between cells and adherent junctions, which limit cell movement and maintain the epithelial laminar arrangement, thereby preserving the structural integrity of the epithelium (11). The integrity of the epithelium mainly depends on adhesion junction proteins (ZO-1, claudins, F11R, E-cadherin) and intracellular proteins (β-catenin) of epithelial cells. These transmembrane proteins adhere to each other by binding between adjacent epithelial cells, and some of them attach to the intracellular actin cytoskeleton, which is essential for maintaining epithelial apical to basal polarity (12, 13). When tissue remodeling occurs, the transmembrane proteins that restrict EMT, from E-cadherin, β-catenin, ZO-1, and occludin, which are unique to epithelial cells, to N-cadherin and α-SMA, which are unique to mesenchymal cells, indicate EMT has occurred (14).

The major known function of the S100A4 protein is to promote tumor metastasis (15). Overexpression of S100A4 in human breast cancer cells promotes lung and lymph node metastasis (16, 17). Inhibition of S100A4 in human bone sarcoma cells and lung cancer cells is associated with reduced metastasis (18, 19). Recently, however, S100A4 has been found to be a powerful factor in various inflammation-related diseases, and the S100 protein family, including S100A4, has been referred to as a part of the damage-associated molecular patterns (DAMPs), which have a key function in the inflammatory reaction in the organism (20, 21). DAMPs are produced and released by damaged and dead cells and can promote inflammation as well as the repair and regeneration of tissues and contribute to the development of a variety of inflammatory diseases, such as metabolic disorders, autoimmune diseases and cancer (22, 23). The pathological processes of tumorigenesis and metastasis are currently considered to be related to a protracted abnormal state of inflammation (24, 25). Accordingly, there are many similarities between chronic inflammatory disease and the molecular processes in cancer development. In chronic inflammatory diseases, the S100 protein family members S100A7, S100A8, S100A9 and S100A12 act as immunomodulators and are significantly associated with the degree of inflammation and tissue remodeling in the disease (26, 27). However, to the best of our knowledge, the regulatory role of S100A4 in CRS tissue remodeling has not yet been reported.

Activation of the Wnt/β-catenin pathway has been suggested to be associated with EMT and β-catenin is involved in the transcription of S100A4 (28, 29), suggesting that S100A4 is involved in the regulatory mechanism of EMT. The canonical Wnt pathway effects through inhibiting GSK-3β-mediated β-catenin hosphorylation and degradation. When Wnt proteins bind to the FZD and LRP5/6 receptors, intracellular DSH is activated by phosphorylation, causing the disassembly of the GSK-3β complex, leading to the accumulation of β-catenin in the cell cytoplasm, and eventually a certain level β-catenin can enter the nucleus (30). After entering the nucleus, β-catenin binds to TCF/LEF to regulate the expression of target genes, including downstream target genes for EMT-related transcription factors, such as SNAIL (31). This study focuses on the process of mesenchymal transformation of nasal mucosal epithelium induced by S100A4 *via* the Wnt/GSK-3β/β-catenin pathway from molecular mechanism to cell morphology, and systematically analyzes the mechanism of CRS tissue remodeling to identify new targets for CRS treatment.

## Materials and Methods

### Subjects

Nine pairs of patients with CRS and matched control subjects with non-CRS-related conditions who attended the Department of Otolaryngology of Shandong Provincial Hospital Affiliated to Shandong First Medical University for functional endoscopic sinus surgery (FESS) (32) or rhinoplasty were recruited for this study. The above-mentioned patients with CRS were diagnosed according to EPOS-2020 criteria (8). All tissues were collected from patients without symptoms of inflammation, allergy, asthma, or aspirin sensitivity. None of the patients had taken oral steroids, nonsteroidal anti-inflammatory drugs, antihistamines, or antibiotics for at least 2 months. Demographic data, Lund-Mackay score and symptom severity score were recorded for each patient. The study was approved by the Ethics Committee of the Affiliated Hospital of Shandong First Medical University (NSFC: No. 2020-354), and written informed consent was obtained from all participants in accordance with the Declaration of Helsinki.

### Liquid Chromatography-Tandem Mass Spectrophotometry (LC‐MS/MS) Analysis and Proteome Analysis

Nasal tissue samples were prepared according to a previously reported protocol (33). Peptides were dissolved in 20 μL of 0.5% TFA and 5% ACN and profiled using a Q Exactive Plus Orbitrap™ mass spectrometer (Thermo Fisher Scientific Inc., Waltham, MA, USA) and separated by liquid chromatography with an EASY-nLC 1000 system (Thermo Fisher Scientific). A binary mobile phase system with 85 min of 0.1% formic acid and 80% acetonitrile plus 0.1% formic acid at a flow rate of 250 nL/min was used for the liquid phase portion. For MS analysis, peptides were loaded onto a 2 cm EASY column precolumn (Thermo Fisher Scientific ID 100 µm, 5 µm, C18) and eluted on a 10 cm EASY column analytical column (Thermo Fisher Scientific ID 75 μm, 3 μm, C18, Thermo Fisher Scientific) for 90 min from 4% to 100% linear gradient of ACN with full scan MS spectra at 70 000 resolution. The top 10 abundant ions were obtained by HCD.

The Uniprot Homo sapiens database (20,199 protein entries) was used for protein identification by comparing the raw data of the peptides using Maxquant (version 1.5.0.1). The search parameters were set to a maximum error tolerance of 10 and 5ppm for survey scanning and MS/MS analysis, respectively. For peptide spectrum matching (PMS) and protein quantification, error detection rate (FDR)was set at 1%. DEPs were tested with right-tailed Fisher exact test (corrected p value < 0.05). The analysis of the DEPs was performed using the Ingenuity^®^ Pathway Analysis (IPA) software (Qiagen, Valencia, CA, USA). We chose the first 10 paths for further analysis. The WEB-based gene set analysis toolkit (WebGestalt) was used for gene set enrichment analysis. The Gene Ontology (GO) and Kyoto Encyclopedia of Genes and Genomes (KEGG) databases were used to perform enrichment and functional analysis of DEPs using hypergeometric tests. Heat maps were plotted using the ClustVis online tool (https://biit.cs.ut.ee/clustvis/).

### Cells Culture

Human nasal epithelial cells (HNEpCs; Cat. No. CBR-130634) were acquired from Cell-Bio Biotechnology Co., Ltd. (Taipei City, Taiwan, China). HNEpCs were maintained in RPMI-1640 (Cat. No. CM1004; Macgene Co., Ltd., Beijing, China) medium at 37°C in 5% CO2 which was supplemented with 10% fetal bovine serum (FBS) (Cat. No. FCS500; ExCell Bio, Shanghai, China) and 1% penicillin-streptomycin (Cat. No. P1400; Sino Biological Inc., Beijing, China).

### Immunohistochemistry Analysis

Immunohistochemistry (IHC) analysis was used to measure the expression of S100A4 and its downstream proteins in samples from CRS patients and healthy control subjects. Briefly, the nasal biopsy tissue was incubated at 60°C for 30 min, deparaffinized with xylene, followed by rehydration with gradient ethanol and then cultured in simmering citrate buffer (Cat. No. C1010; Solarbio Science & Technology Co., Ltd., Beijing, China) for 10 min to complete antigen repair. After treatment with perhydrol for 20 min, the specimens were blocked with 5% bovine serum albumin (BSA). Then, individual tissue slides were separately incubated at 4°C overnight with the corresponding primary antibody, including the following: S100A4 antibody (Cat. No. A1631; 1:200; ABclonal Technology, Woburn, MA, USA), COL1A1 antibody (Cat. No. ab34710; 1:200; Abcam, Cambridge, UK), β-catenin antibody (Cat. No. 8480S; 1:100; Cell Signaling Technology (CST), Danvers, MA, USA), E-cadherin antibody (Cat. No. 20874-1-AP; 1:500; ProteinTech Group, Rosemont, IL, USA), and α-SMA antibody (Cat. No. bs-10196R; 1:200; Bioss Antibodies, Woburn, MA, USA). Subsequently, the tissue slides were incubated for 1 h with the appropriate goat anti-rabbit labeled secondary antibody (Cat. No. ZB-2301; 1:200; ZhongShan Golden Bridge (ZSGB)-BIO, Beijing, China) and immunoreactivity was eventually detected using a diaminobenzidine (DAB) horseradish peroxidase chromogenic kit.

### Immunofluorescence

After washing with cold phosphate-buffered saline (PBS), HNEpCs were directly fixed with 4% polyformaldehyde (Cat. No. P0099; Beyotime Biotechnology, Shanghai, China) and then permeated with 0.3% Triton X-100 (Cat. No. T8200, Solarbio Science & Technology Co., Ltd.) in PBS. The non-specific sites of the samples were blocked with sheep serum and the cells were then stained with a primary anti-S100A4 antibody (Cat. No. A1631; 1:200; ABclonal Technology) overnight at 4°C. The samples were then rinsed with PBS and incubated with fluorescently labeled goat anti-rabbit antibody at room temperature for an additional 1 h. Afterwards, a 4′,6-diamidino-2-phenylindole (DAPI) dye solution (Cat. No. C0060; Solarbio Science & Technology Co., Ltd.) was added and incubated for 10 min in the dark. Finally, the nuclei were counted, and the stained cells were analyzed under a fluorescent microscope.

### Western Blot Analysis

Total protein was extracted from HNEpCs or frozen nasal tissues with RIPA lysis buffer (Cat.No.P0013B; Beyotime Biotechnology). The nuclear proteins and cytoplasmic proteins of LPS-stimulated HNEpCs were extracted and separated using the Nuclear and Cytoplasmic Protein Extraction Kit (Cat. No. P0027; Beyotime Biotechnology) and quantified using the bicinchoninic acid (BCA) Protein Assay kit (Cat. No. P0010S; Beyotime Biotechnology). Subsequently, 50 µg of protein samples were loaded and separated by 10 and 8% sodium dodecyl sulfate‐polyacrylamide gel electrophoresis (SDS‐PAGE) and transferred onto polyvinylidene difluoride membranes (PVDF). After blocking the membranes with 5% fat-free milk for 1 h, followed by overnight incubation at 4°C with the followin individual mouse monoclonal antibodies: S100A4 antibody (Cat. No. A1631; 1:1,000; ABclonal Technology), COL1A1 antibody (Cat. No. ab34710; 1:5,000; Abcam), GSK-3β antibody (Cat. No. 12456S; 1:1,000; Cell Signaling Technology), β-catenin antibody (Cat. No. 8480S; 1:1,000; Cell Signaling Technology), E-cadherin antibody (Cat. No. 20874-1-AP; 1:5,000; ProteinTech Group), and α-SMA antibody (Cat. No. bs-10196R; 1:5,000; Bioss Antibodies) and the internal reference rabbit antibody GAPDH (Cat. No. TA-08; 1:1,000; ZSGB-BIO). Next day, after washing with PBS, the membranes were incubated at 37°C with the appropriate secondary antibody, including anti-mouse IgG (Cat. No. zb-2301; 1:2,000; ZSGB-BIO) and anti-rabbit IgG; (Cat. No. zb-23051; 1:2,000; ZSGB-BIO). Subsequently, after washing with tris-buffered saline containing Tween 20 (TBST) buffer, the signals were visualized using an enhanced chemiluminescence imaging system (Cat. No. Tanon-4600; Tanon Science & Technology Co., Ltd, Shanghai, China).

### Epithelial-Mesenchymal Transition (EMT) Induction and Assessment

To induce EMT, the cells were incubated with 1, 2 and 4 ug/ml LPS (Cat. No. L8880; Solarbio Science & Technology Co., Ltd.) for 6 and 24 h, respectively. The morphology of the cells was observed by phase contrast microscopy. The protein expression levels of E-cadherin and α-SMA were determined by Western blot analysis, as described above.

### RNA Interference and Transfection

The small interfering RNAs (shRNAs) targeting S100A4 (sh-S100A4) were obtained from Shanghai GenePharma Co., Ltd (Shanghai, China). Three sequences of the sh-S100A4 were used, and in order to improve the transfection efficiency, HNEpCs were transfected twice. Briefly, after the confluence reached 30%, HNEpCs were transfected with 30 nM shRNA using Lipofectamine 2000 (cat. No. 11668030; Invitrogen, Carlsbad, CA, USA) following the manufacturer’s instructions. After 24 h of culture, the second transfection was performed. Cells were then cultured for 24 h and collected. The sh-S100A4 sequences are as follows:

sh-S100A4-1 TGGGCTTGCACACGCTGTTGCTATA

sh-S100A4-2 GCTTGCACACGCTGTTGCTATAGTA

sh-S100A4-3 CGCTGTTGCTATAGTACGTGTTGAT

negative control (nc) TTCTCCGAACGTGTCACGT

### Cells Migration Scratch Assay

The cells were seeded in 6-well plates and cultured at 37°C until 80% confluence was reached. A scratched wound was created with the tip of the pipette, then the cells were washed with PBS, and serum-free medium was added to each well. After 24 h of culture, the same area of each wound was captured with an inverted microscope (Cat. No. XD-202; Nanjing Jiangnan Yongxin Optics Co., Ltd., Nanjing, China), and the relative mobility of cells in each group was calculated.

### Transwell Migration Assay

The cells were seeded in the upper part of the Transwell chamber. Briefly, a 300 µL aliquot of cell suspension (containing about 5,000 cells) was added to each well of the upper chamber, and 500 µL of complete serum-containing medium is added into each well of the lower chamber. After incubation for 24 h, the cells on the top surface of the membrane were removed and the cells on the bottom surface of the membrane were stained with 0.1% crystal violet (cat. No. g1064; Solarbio Science & Technology Co., Ltd.). Images of stained were acquired using a microscope at 400 × magnification, from five high-power fields (HPFs) and migrated cells were counted.

### Chromatin Immunoprecipitation

For chromatin immunoprecipitation (ChIP) assay, 5 × 10^^6^ HNEpCs were cross-linked with formaldehyde and lysed, with IP lysis buffer (Cat. No. G2038; Servicebio Technology Co., Ltd., Wuhan, China) containing protease inhibitor (Cat. No. G2007, Servicebio Technology Co., Ltd.) by subjecting the cells to ultrasonic lysis until the average DNA fragment size was 1,000 bp. Then, 90 μL of cleared lysate was kept for the input test, 40 μL of the product was added to 10 μL of 5* reduced protein sampling buffer (Cat. No. G2013, Servicebio Technology Co., Ltd.), denatured by heating and subjected to Western blot analysis detection, and the DNA in the remaining 50 μL of the product was uncrosslinked from protein overnight and used for polymerase chain reaction (PCR) amplification. The effect of ultrasonic fragmentation was evaluated and the presence of target DNA and protein in the sample was confirmed. Subsequently, 100 μL of the product was diluted in 900 μL of ChIP dilution buffer containing 1 mM phenylmethylsulfonyl fluoride (PMSF; Cat. No. G2008; Servicebio Technology Co., Ltd.) and 20 μL of 50* cocktail (Cat. No. G2006, Servicebio Technology Co., Ltd.). Then, cleared lysates were precleared with bovine serum albumin (BSA; Cat. No. G2026, Servicebio Technology Co., Ltd.)salmon sperm DNA blocking protein A/G plus agarose (Cat. No. IP05, Millipore, Burlington, MA, USA) and incubated overnight at 4°C with corresponding primary antibodies. Afterwards, immune complexes were captured with BSA/salmon DNA blocking protein A/G plus agarose, washed, and the beads were resuspended in TE buffer. The centrifuged solution was added to a CB3 adsorption column for purification. The transcription factor binding site was predicted using known transcription factor binding site motifs in the Jaspar database. Primers for PCR screening were designed and synthesized according to the binding site. The antibodies used for ChIP were anti-β-catenin (Cat. No. 8480S; Cell Signaling Technology) and anti-TCF-4 (Cat. No. ab217668; Abcam).

### Co-Immunoprecipitation (Co-IP)

Cells were collected by centrifugation with cold IP cell lysis buffer (Cat. No. G2038; Servicebio Technology Co., Ltd.) and incubated at 4°C for 10 min, and then centrifuged at 12,000 g for 10 min. The protein content of the supernatant was determined by the BCA method. A small aliquot of supernatant was taken for the input experiment after denaturation. Then, the β-catenin antibody, TCF-4 antibody and inactive rabbit IgG were added at a concentration of 1 µg/mL and incubated overnight at 4°C on a shaking bed. On the second day, a 80-µL aliquot of protein A/G-beads (Cat. No. IP05; Millipore) was added to the tube and shaken slowly at 4°C for 2 h. The supernatant was removed after centrifugation, and the precipitate was washed 4 times with 1 mL of cold IP lysis buffer 4 and centrifuged. After the last wash, the supernatant was discarded, and the precipitate was resuspended in 80 µL of 1× SDS-PAGE sample loading buffer and boiled for 10 min. Eventually, a 10-μL aliquot of supernatant was centrifuged for Western blot analysis detection.

### Statistical Analysis

Statistical analysis was conducted using the SPSS (v.19.0) software (IBM Corporation, Armonk, NY, USA. All data were expressed as the mean ± standard deviation. The significant difference between groups was evaluated using an independent t-test or analysis of variance (ANOVA). All experiments were performed at least three times, and P < 0.05 was considered as a statistically significant difference.

## Results

### Proteome Comparison Between Nasal Mucosal Tissues of CRS Patients and Control Subjects

**Figure 1:**
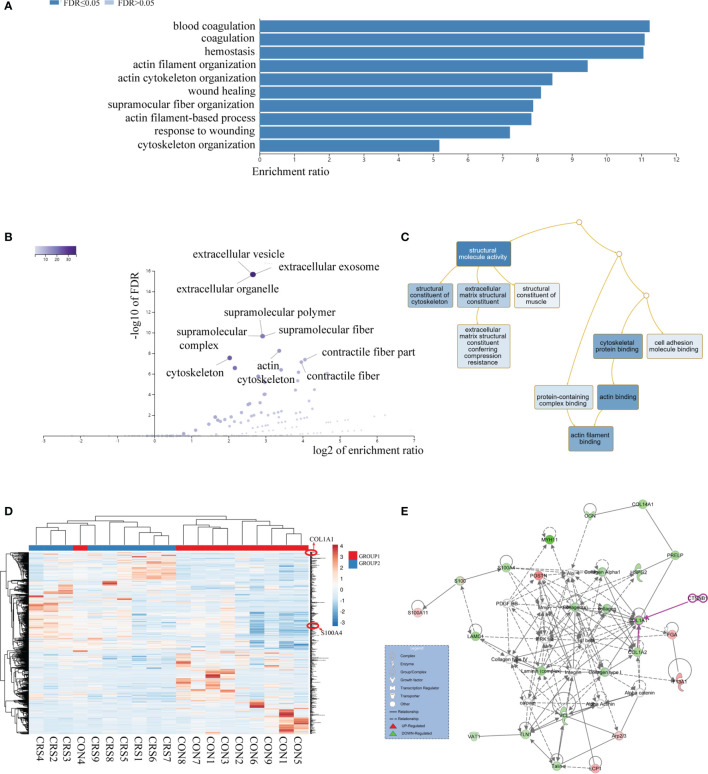
Proteome comparison between nasal mucosal tissues of CRS patients and control subjects. **(A)** The biological process (BP) analysis of genetic ontology (GO) of differential proteins. **(B)** Analysis of cellular composition (CC) of genetic ontology (GO) of differential proteins. **(C)** Molecular functional (MF) analysis of genetic ontology (GO) of differential proteins. **(D)** Heatmap analysis of differential proteins. HP, CRS patient samples; HT, Healthy control samples. **(E)** Network analysis of differential proteins. Each circle represents a protein, and the lines between proteins represent interactions between them.

A total of 2,753 proteins were identified and quantified, and 69 proteins were significantly differentially expressed between the nasal mucosa of CRS patients and normal control subjects. Among them, 44 proteins were significantly increased and 25 proteins were decreased in CRS patients (**Table 1**) (P < 0.05). The GO classification system revealed the biological significance of the different proteins (**Figures 1A–C**). Most of the proteins were contractile fibers and actin cytoskeleton components, and their molecular functions included extracellular matrix structural constituent and cytoskeleton structure. They were involved in various biological processes, including cytoskeleton organization and wound healing. The changes in intercellular structure, enhanced cell movement, and extracellular collagen deposition and tissue remodeling occurred in CRS, which suggested that EMT may occur in CRS. In addition, the heatmap analysis of the expression of differential proteins using ClustVis revealed (**Figure 1D**) that the DEPs were mainly members of the calcium-binding protein family (S100) and collagen protein family (COL). As shown in **Figure 1E**, the IPA network analysis indicated that the COL family and the S100 family of DEPs interacted and correlated with the β-catenin signaling pathway. Studies have shown that S100A4 is associated with pleural mesothelial cells and EMT in biliary atresia (32, 33). And COL1A1 is associated with EMT in breast cancer (34). Therefore, we speculate that S100A4 and COL1A1 in CRS may be involved in the EMT process of nasal mucosal epithelial cells *via* the Wnt/β-catenin signaling pathway.

**Table 1:** Proteins with significant differences in chronic rhinosinusitis.

Gene names	Protein IDs	Ratio N/CRS	P value
H2AFV;H2AFZ	Q71UI9;P0C0S5	0.510863031	0.000124535
HEL-76;CA2	V9HW21;P00918	0.308414813	0.000762733
EHD2	A0A024R0S6	0.467713904	0.000827173
ACTC1;ACTA1	P68032;A8K3K1	0.446532834	0.001300552
COL1A1	P02452;Q6LAN8	0.38986831	0.002298073
HSPG2	A0A024RAB6	0.516110083	0.002638246
TMSB4X	A2VCK8;P62328	1.602381543	0.003063176
HEL32;DSTN	V9HWA6;P60981	0.60563386	0.003155291
COL1A2	A0A087WTA8	0.492639727	0.003260182
MYH11	A0A024QZJ4	0.212257392	0.003346806
HEL-S-37;LCP1	V9HWJ7	1.980555364	0.003650284
SPTBN1	B2ZZ89;Q01082	0.628945443	0.00383177
DPYSL2	Q59GB4;Q16555	0.666200395	0.004132201
SPTAN1;DKFZp564P0562	A0A024R889	0.624917927	0.004580224
VCL;HEL114	A0A024QZN4	0.57391272	0.004628198
S100A4	P26447	0.567163161	0.00592066
ACTR3	A0A024RAI1	1.300310678	0.00632945
HEL-S-43;S100A11	V9HWH9	1.412661446	0.008154354
TPM1	Q6ZN40	0.389343295	0.00909404
TPP1	B4DSE2;B4E0C7	2.081267391	0.00912802
HEL-S-30;PKM;PKM2	V9HWB8;P14618	1.243313331	0.010045356
FLNA	Q5HY54;P21333	0.560536843	0.010739654
LMNA	P02545;Q5I6Y5	0.748100228	0.010997514
POSTN	A0A024RDS2	2.823890603	0.011088399
ITIH2	Q5T985;A2RTY6	2.249616312	0.011570922
TLN1	Q9Y490	0.672497924	0.011750335
PRELP;MST161	Q6FHG6;P51888	0.525103605	0.011763869
LAMC1	A0A024R972	0.456545543	0.012328024
PTRF	B4DPZ5;B4DNU9	0.53049269	0.012414864
F13A1	P00488;B2R6V9	2.2500092	0.013013469
ADH5	Q6IRT1;Q6FI45	0.768391559	0.013278048
VAT1	A0A024R1Z6	0.744177822	0.014065971
HEL-S-273	V9HW25	0.497114864	0.014701114
A8K008_HUMAN Uncharacterized	A8K008	2.167693738	0.016541779
GLUD1;GLUD2	B4DMF5	0.730647486	0.017498562
CSRP1;DKFZp686M148	B4DY28;P21291	0.450650197	0.017815294
LMNB1	P20700;E9PBF6	1.915906076	0.017880373
ACTR2	P61160;Q8IY98	1.355676164	0.017910762
SH3BGRL3;HEL-S-297	Q5T123;Q86Z22	1.556469071	0.018393109
HEL-S-34;PEBP1	D9IAI1;P30086	0.652088077	0.019168704
CORO1A	A0A024R611	2.537679806	0.020117333
HEL107;TKT	V9HWD9	1.628929183	0.020275063
CP	A8K5A4;A5PL27	5.147710911	0.021794028
OGN	A8K0R3;B4DI63	0.563131206	0.023578264
KRT5	CON:P13647	1.923428112	0.023694742
COL1A1	D3DTX7	0.55576631	0.024898639
ARHGDIB	A0A024RAS5	1.695863555	0.025300843
MYL6	F8W1R7;G3V1V0	0.675981091	0.025402265
CBR1	P16152	0.717892593	0.026627938
KCTD12	B3KY04	0.708919	0.027680969
HBA2;HBA1	D1MGQ2	0.616303185	0.028224531
TAGLN	Q5U0D2	0.557283225	0.02879555
TPM3;DKFZp686J1372	A0A0S2Z4G4	1.224835548	0.029416277
IGL@	Q8N355	3.04147209	0.03074476
GGT5	B4DND4;P36269	0.675807813	0.032241336
KRT15	CON:P19012	2.520130695	0.034869703
PSAP	Q53FJ5	1.367275834	0.035510117
COL14A1	Q05707;A8KAL5	0.482138598	0.035549304
hCG_40889;CFH	A0A024R962	2.384107014	0.03647054
UQCRC2	P22695;H3BRG4	0.73400239	0.03963777
SOD3	A0A140VJU8	0.479436212	0.04003326
FGA	P02671	1.881004106	0.040339534
CAPZB	B2R7T8;P47756	1.224661199	0.040612013
RPS18	P62269	0.746554987	0.044336416
YWHAZ	D0PNI1;P63104	0.831646141	0.044958897
MYL12B	O14950	0.69090751	0.045432789
DPYSL3	A0A140VK07	0.731155232	0.046165319
KRT8	CON:P05787	0.676382105	0.048060684
HEL-S-123m;ATP5A1	V9HW26;P25705	0.77635782	0.048791926

### The Expression of S100A4 and COL1A1 Is Increased in CRS Nasal Mucosa

**Figure 2:**
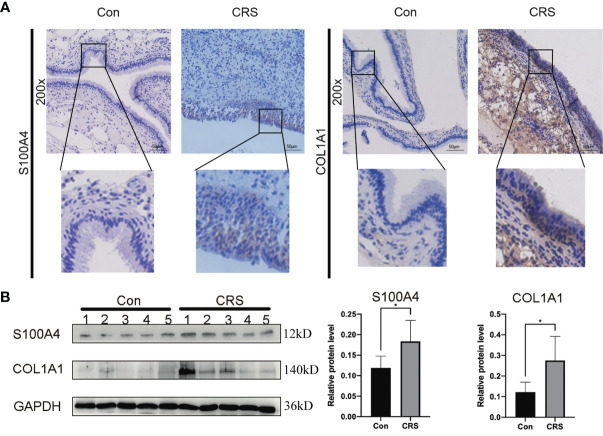
Expression of S100A4 and COL1A1 in tissues of chronic rhinosinusitis group. **(A)** The expression of S100A4 and COL1A1 proteins in chronic sinusitis tissues and normal control tissues were detected by immunohistochemistry. The upper image is at 200X magnification, and the lower image shows a partially magnified image in a black box.bar=50um. **(B)** Western blot was used to detect the expression of S100A4 and COL1A1 proteins in chronic rhinosinusitis tissue and normal control tissue. The bar graph on the right shows the protein expression statistics. *P < 0.05.

The results of the IHC analysis of the protein expression level of S100A4 and COL1A1 in the nasal mucosa of CRS patients revealed that the protein levels of S100A4 and COL1A1 were significantly higher in the CRS group than in the control group. The S100A4-positive cells had brown-yellow cytoplasm or nucleus, and the color was mainly observed in the mucosal epithelium and glandular epithelium (**Figure 2A**). The COL1A1-positive cells had mainly a brownish color in the cytoplasm and extracellular connective tissue. The evaluation of the changes in S100A4 and COL1A1 protein levels in the total protein of nasal mucosal tissue of the CRS group and the control group by Western blot analysis further confirm the dysregulation of the expression of S100A4 and COL1A1 in CRS. These findings indicated that the expression of both S100A4 and COL1A1 were clearly increased in the CRS group compared with the control group (**Figure 2B**).

### Expression of EMT Markers in Nasal Mucosa of CRS Patients and Control Subjects

**Figure 3:**
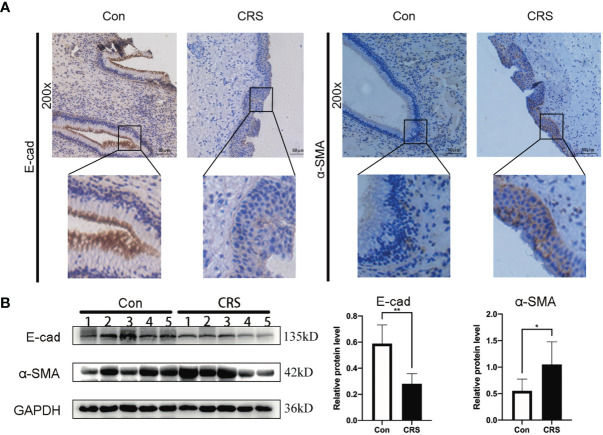
Expression of EMT markers in chronic rhinosinusitis tissues. **(A)** The expression of markers of EMT in chronic sinusitis tissues and normal control tissues were detected by immunohistochemistry. The upper image is at 200X magnification, and the lower image shows a partially magnified image in a black box.bar=50um. **(B)** Western blot was used to detect the expression of markers of EMT proteins in chronic rhinosinusitis tissue and normal control tissue. The bar graph on the right shows the protein expression statistics. *P < 0.05, **P < 0.01.

We previously hypothesized, based on bioinformatics, that S100A4 and COL1A1 might be involved in the EMT process of CRS. To determine whether nasal mucosal epithelial cells of CRS tissue can transform into mesenchymal cells, we evaluated the expression level of E-cadherin and α-SMA in the CRS and control groups. IHC analysis results revealed that the expression of E-cadherin protein was decreased in the CRS group compared with the control group, while the expression of α-SMA protein was significantly increased (**Figure 3A**). In addition, these findings were confirmed by Western blot analysis (**Figure 3B**). Thus, these results indicated that epithelial cells can transform into mesenchymal cells in CRS tissues.

### LPS Induces S100A4 and EMT Markers Expressions in HNEpCs

**Figure 4:**
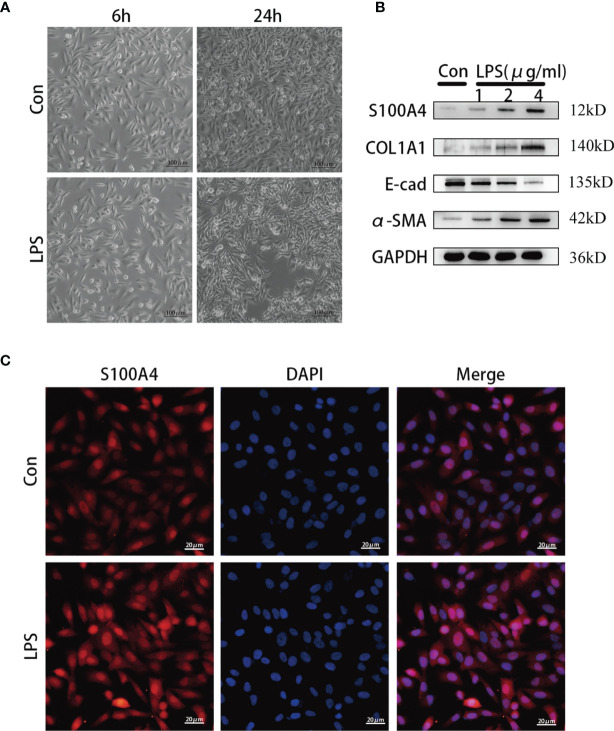
The expression of S100A4 and COL1A1 increased during the process of epithelial-mesenchymalization of nasal mucosa epithelial cells. **(A)** The morphology of HNEpC cells after LPS stimulation for 6h and 24h under phase contrast microscope.bar=100um. **(B)** Western blot was used to detect the expressions of S100A4, COL1A, α-SMA and E-cad in HNEpC cells stimulated with different concentrations of LPS (1-4ug/ml) for 24h. **(C)** Immunofluorescence was used to observe the expression of S100A4 in the nucleus and cytoplasm of HNEpC cells stimulated by LPS for 24 h. Red represents S100A4 and blue represents nuclear staining (DAPI). bar=20um.

To simulate the inflammatory conditions *in vitro*, HNEpCs were stimulated with LPS, and the mesenchymal phenotype of HNEpCs was observed by phase contrast microscopy. After 24 h of LPS stimulation, epithelial cells began to become flat and extend in opposite directions, showing the typical spindle shape of fibroblasts (**Figure 4A**). In addition, Western blot analysis (**Figure 4B**) showed that with the increase of the LPS concentration the protein expression of S100A4, COL1A1 and α-SMA increased gradually, while the protein expression of E-cadherin decreased. Moreover, immunofluorescence staining revealed that S100A4 protein was present in the cytoplasm and nucleus of HNEPCs, and LPS stimulation increased the expression of S100A4 protein (**Figure 4C**).

### Downregulation of S100A4 Reverses the EMT of HNEpCs

**Figure 5:**
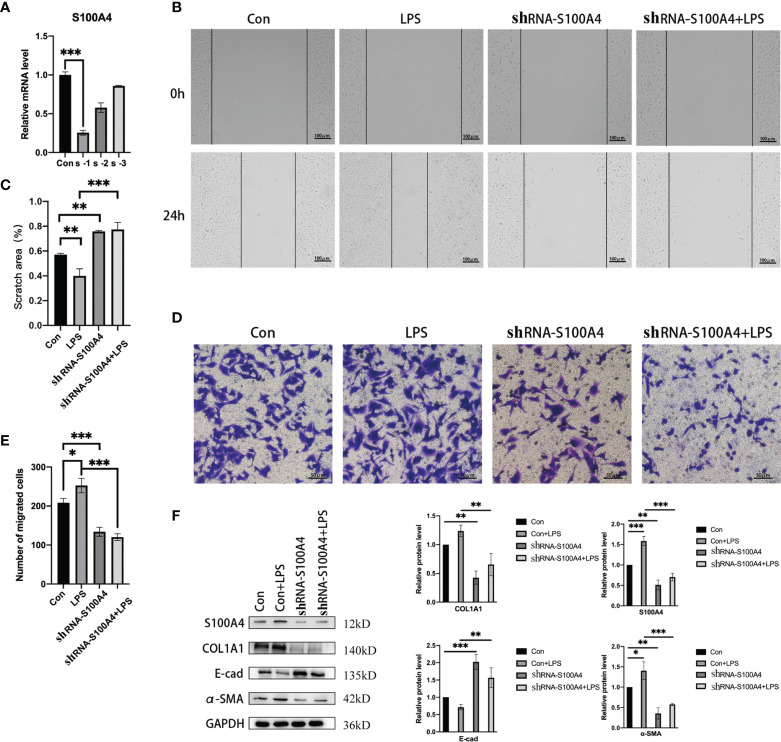
Epithelial mesenchymal changes in HNEpC cells after knockdown of S100A4. **(A)** RT-PCR was used to detect the knockdown efficiency of shRNA-S100A4 transfected HNEpC cells. **(B)** After LPS treatment of HNEpC cells for 24 h or transfection with shRNA-S100A4, cell scratch assay was used to detect cell migration. **(C)** Statistics of cell scratch area. **(D)** Transwell assay for cell migration. **(E)** Statistical graph of the number of migrated cells. **(F)** Western blot detection of epithelial-mesenchymal-related protein expression in HNEpC cells after S100A4 knockdown. Histograms on the right represent protein expression statistics. *P < 0.05, **P < 0.01, ***P < 0.001.

The enhancement of migration ability is one of the important functional characteristics of mesenchymal cells. We confirmed that S100A4 overexpression is associated with EMT in HNEpCs, thus we used shRNA against S100A4 to downregulate its expression (**Figure 5A**) and eventually selected the sh-S1004A-1 group for follow-up experiments to evaluate the EMT of HNEpC by observing their migration ability. As shown by the cell migration scratch assay (**Figures 5B, C**), compared with the control group, the cell scratch area in the LPS-stimulated group was significantly reduced, while in the sh-S100A4-1 group, the cell scratch area was significantly increased (P <0.05). Furthermore, as shown by the transwell migration assay (**Figures 5D, E**), the migrating cell count increased to 250 in the LPS-stimulated group compared with the control group. After downregulating S100A4 with sh-S1004A-1, these effects could be reversed, and the number of migrated cells decreased to about 130. The above findings were confirmed by the Western blot analysis of the protein level changes of S100A4, COL1A1, E-cadherin and α-SMA in the four groups (**Figure 5F**). The Western blot analysis results showed that the stimulatory effects of LPS on the expression of COL1A1 and α-SMA proteins were abolished, while the expression of E-cadherin protein was increased when the S100A4 expression was downregulated by sh-S1004A-1.

### S100A4 Expression Is Regulated *via* the Wnt/GSK-3β/β-Catenin/TCF-4 Signaling Pathway in CRS

The above IPA network analysis results showed that S100A4 was closely related to the EMT-associated Wnt/β-catenin signaling pathway. In combination with a literature review, we found that the half-life of β-catenin in the cytoplasm is very short (35–37). It is inactivated after phosphorylation by a “destruction complex” composed of GSK-3β, CK-Iα, AXIN and APC. In contrast, in response to specific signaling stimulation, mutation of a critical phospho-site in the “destruction complex”, the β-catenin protein appears to accumulate in the cytoplasm, followed by nuclear translocation. In tumor cells, the T-cell factor (TCF) binding motifs activate the transcription of the target gene S100A4 (31, 38). Therefore, we suspect that direct targeting binding of S100A4 to the β-catenin/TCF complex in CRS regulates the Wnt/β-catenin signaling pathway, resulting in abnormal expression of the downstream EMT-related protein COL1A1, ultimately leading to tissue remodeling in the nasal mucosa.

**Figure 6:**
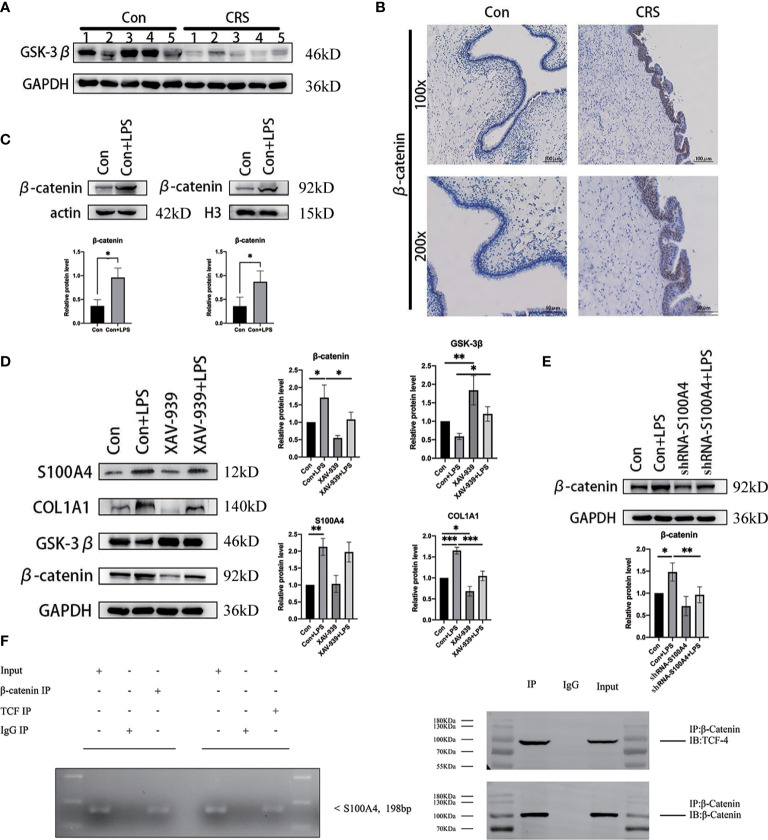
S100A4 is a direct transcriptional target of the Wnt/β-catenin/TCF-4-mediated signaling pathway, encoding the protein COL1A1. **(A)** Western blot detection of GSK-3β protein expression in chronic sinusitis tissue. **(B)** Immunohistochemical observation of the expression of β-catenin in chronic sinusitis tissue. Image magnification: 100×, bar=100um and 200×, bar=50um. **(C)** Western blot was used to detect the expression of β-catenin protein in the nucleus and cytoplasm of HNEpC cells after LPS stimulation, below is a graph of protein expression statistics. **(D)** The expressions of S100A4, COL1A1 and GSK-3β after β-catenin inhibition were detected by Western blot. Shown on the right is a statistical graph. **(E)** Detection of β-catenin protein expression in HNEpC cells after S100A4 knockout. **(F)** ChIP for binding of β-catenin and TCF-4 to the S100A4 promoter in LPS treated HNEpC cells, S100A4-specific PCR products were amplified following ChIP with TCF-4 antibody, as well as from the input of ChIP assay, while S100A4-specific PCR product was not detected in immunoglobulin G control. Co-IP results showed that β-catenin could be detected in the immune complex pulled by TCF-4 antibody, while β-catenin and TCF-4 proteins could not be detected in the immune complex pulled down by ineffective IgG. *P < 0.05, **P < 0.01, ***P < 0.001.

We first performed Western blot analysis on CRS and normal nasal mucosal tissues (**Figure 6A**), and the results showed that expression of GSK-3β protein, a component of the destruction complex, was significantly decreased in CRS. Afterwards, we examined β-catenin protein expression in CRS tissues by IHC analysis and found that β-catenin protein expression was markedly increased in the CRS group compared with the control group (**Figure 6B**). Western blot analysis of the cytoplasmic and cytosolic proteins from LPS-stimulated HNEPCs revealed that the expression of β-catenin in both cytoplasm and nucleus was higher than that in the control group (**Figure 6C**).

To further confirm whether S100A4-induced EMT is regulated by β-catenin, we treated HNEpCs with the β-catenin inhibitor XAV-939 to study the effect of downregulating β-catenin on the degree of expression of S100A4 and COL1A1 (39). The Western blot analysis results revealed that compared with the control group, the expression levels of S100A4, COL1A1 and β-catenin proteins were increased in the LPS-stimulated group, while the expression of GSK-3β protein was decreased. These results show that inflammation stimulates nasal mucosal epithelial cells, leading to increased expression of S100A4 n and β-catenin of the Wnt pathway and COL1A1 a protein downstream of EMT. Compared with the LPS-stimulated group, after GSK-3β overexpression in the XAV-939+LPS group, the expression levels of β-catenin, COL1A1 and S100A4 proteins showed a decreasing trend (**Figure 6D**). Conversely, analysis of the expression of β-catenin protein after knocking down S100A4 with sh-S100A4 revealed that compared with the LPS-stimulated group, the expression of β-catenin was decreased in the si-S100A4+LPS-stimulated group (**Figure 6E**). This finding suggests that β-catenin may interact with S100A4 to induce the overexpression of COL1A1, a protein downstream of EMT.

**Figure 7:**
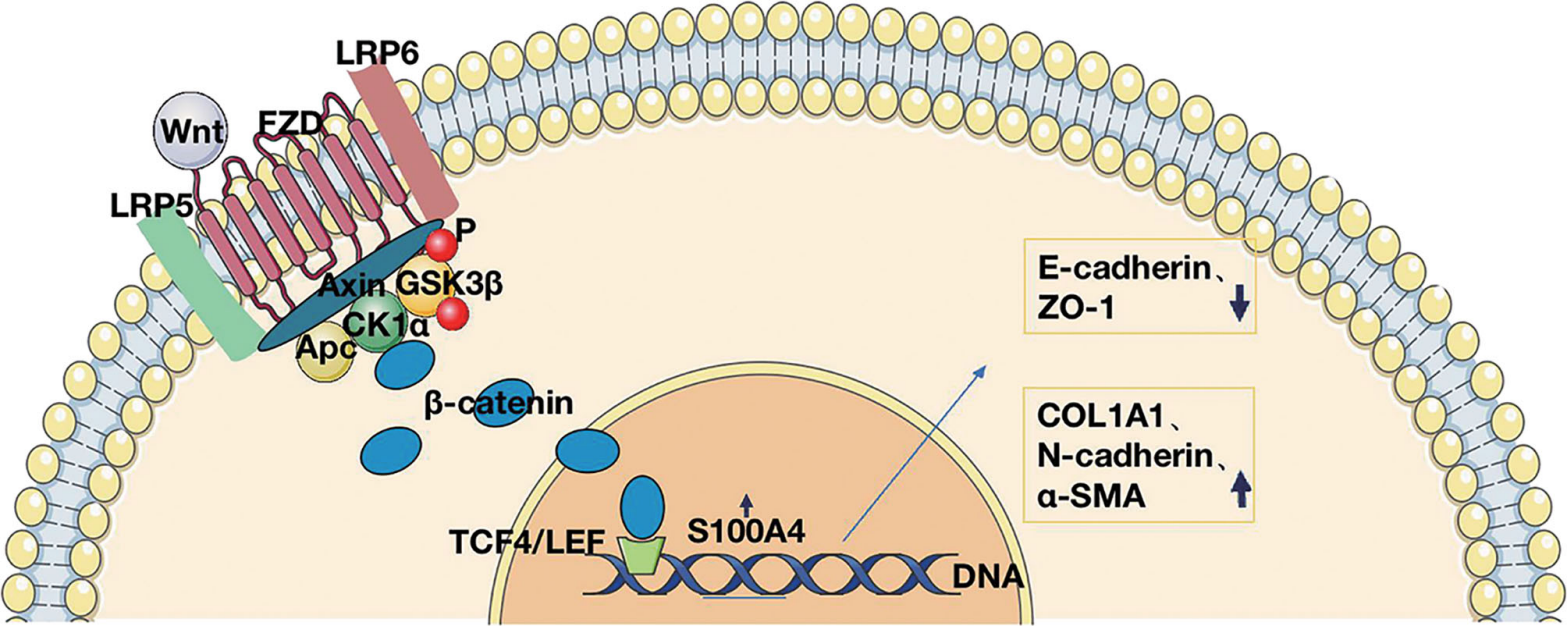
Schematic representation of S100A4 regulation of the Wnt/β-catenin/TCF-4 pathway in CRS.

Also, to further establish whether S100A4 is a direct target of β-catenin, we analyzed the sequence of the gene from the human S100A4 promoter and found a TCF-4 binding site sequence. The binding of the transcription factor TCF-4 to the S100A4 promoter was confirmed by the results of the CHIP assay (**Figure 6F**). Given all the above data, we conclude that TCF-4 can specifically bind β-catenin from cellular immunoprecipitates, which shows that S100A4 is directly regulated *via* the Wnt/GSK-3β/β-catenin/TCF-4 pathway, which is involved in regulating the specific protein COL1A1 related to mesenchymal cell morphology and cell movement (**Figure 7**).

## Discussion

This study describes the identification, by proteomics and bioinformatics approach, of the differential protein expression of S100A4 in CRS. It targets the downstream transcription factor TCF-4 of the Wnt signaling pathway and induces the expression of the downstream mesenchymal protein COL1A1, thereby promoting EMT in the nasal mucosa. The exosome proteome revealed that the human nasal epithelium-derived secreted exosomes from Chronic rhinitis sinusitis with nasal polyps (CRSwNP) patients contain proteins that affect cell proliferation pathways and are involved in epithelial remodeling, mainly *via* p53-mediated pathways and other pathways, which may lead to remodeling of the sinus mucosa (40). Both, the nasal mucus proteome and mucosal proteome, suggest that CRS is associated with tissue remodeling (9, 10). In this study, we performed mass spectrometry analysis of the nasal mucosal proteome and identified 2,753 proteins expressed in CRS tissues. Among them, we found several DEPs, including collagen family proteins, such as COL1A1, COL1A2 and COL14A1, and S100 family proteins, such as S100A4 and S100A11, which have functions related to tissue remodeling, such as contractile fibers, actin cytoskeleton, muscle structural architecture and cytoskeletal structural architecture. Our proteomics identified DEPs in CRS that are involved in the tissue remodeling process. We used mass spectrometry analysis combined with bioinformatics to innovatively identify S100 protein that plays an important role in tissue remodeling. Additionally, we investigated the regulatory mechanism of S100A4 in nasal mucosal tissue remodeling during CRS.

There are three subtypes of EMT depending on the biological context of its function: type 1, which is associated with physiological processes, such as embryology, development and organ formation; type 2, which is associated with tissue regeneration, damage repair and fibrosis; type 3, which is associated with malignant tumor invasion (41). Accordingly, the functional subtype of EMT in CRS is mainly type 2 EMT. Under physiological conditions, when the inflammatory response is relieved, the EMT process will gradually stop on its own (42, 43). However, if the nasal inflammatory response continues to be activated, likewise, EMT will persist, eventually causing massive collagen deposition in the nasal mucosa and ultimately irreversible tissue remodeling of the nasal mucosa (44). In fact, numerous studies have confirmed that S100A4 is involved in EMT during inflammation (45). However, whether S100A4 causes EMT in CRS has not been demonstrated. In this study, we showed that high expression of S100A4 in CRS was accompanied by significant changes in EMT marker proteins and cell morphology as well as cell motility *in vitro* and *in vivo*. On the other hand, we observed a reversal in the expression of EMT marker proteins in nasal mucosal epithelial cells after inhibiting the expression of S100A4, which demonstrated that S100A4 is a key component of the EMT process in CRS.

S100A4 has different intracellular and extracellular activities. In cells, the expression of S100A4 is related to cell migration and the maintenance of stem cell stability (46, 47). In terms of regulating cell migration, intracellular S100A4 interacts with rhotekin, promyosin and actin to regulate cell migration and invasion (48). S100A4 interacts with thioredoxin leading to intracellular microfilament remodeling and alterations in cell motility and adhesion (49). The proteomics analysis in this study identified DEPs associated with cell migration, including myosin light polypeptide 6, ACTB, type II cytoskeletal 8. The S100A4 protein in CRS may interact with such proteins to increase the motility of nasal mucosal epithelial cells, but this needs to be further investigated. However, our scratch migration assay and transwell assay revealed that S100A4 overexpression in nasal mucosal epithelial cells in an inflammatory environment could enhance the motility of nasal mucosal epithelial cells. This suggests that S100A4 can promote the migration of nasal mucosal epithelial cells in CRS. In addition, S100A4 exerts its effect outside the cell by triggering the hyperinflammatory immune response and various factors, such as cytokines, extracellular matrix (ECM) proteins and matrix metalloproteinases (MMPs) (50–53). There is growing evidence that increased levels of S100A4 in the exocytic sphere are significantly associated with various human inflammatory indications (54–56). S100A4 also stimulates the secretion of hyperinflammatory agents, including cytokines (25). Cytokine-mediated pathways can stimulate the secretion of S100A4 by various cells, such as IL-7-mediated secretion of S100A4 (57). LPS is present in the cell wall of Gram-negative bacteria and bacterial infection can trigger a range of inflammatory responses mediated by a variety of inflammatory cell types (58, 59). And many studies have shown that LPS can promote EMT in cells (60, 61). Therefore, we stimulated HNEpC cells with LPS to establish an EMT model of nasal mucosa epithelial cells. In this study, we found increased expression of S100A4 and overexpression of COL1A1, as well as changes of EMT marker proteins after stimulation of HNEpCs with LPS, suggesting that S100A4 plays an important role in the pro-inflammatory pathway and causes extracellular collagen deposition, which is a major component of the ECM. Furthermore, the specific expression of S100A4 in CRS leads to the remodeling of the ECM. On the one hand, S100A4 processing in fibroblasts leads to the induction of the secretion of collagen (62, 63), which is a major component of the ECM. On the other hand, the ECM makes a difference in supporting immunological reactions at the affected site due to changes in the cytokine pool during the inflammatory process. Cells at the site of the lesions receive cytokines associated with S100A4 in the ECM (64). In addition, the activity of the S100A4 protein stimulates the structural molecular changes of the ECM and the production of MMPs related to ECM remodeling, which affect the process of ECM remodeling by degrading the proteins in the ECM (65–68). Moreover, S100A4 as a fibroblast-specific protein is associated with tissue remodeling and inflammatory cell recruitment in CRS studies and increased fibroblasts are associated with decreased quality of life in patients with CRSwNP and allergic fungal rhinosinusitis (AFRS) (69). However, the S100A4-induced process of nasal mucosal remodeling has not been fully explained in studies to date. In this study, based on the observation of morphological changes after LPS stimulation of HNEpCs and enhanced cell migration function, it is suggested that the process of tissue remodeling that occurs in CRS is closely related to EMT.

We analyzed the S100A4 expression regulation network and found that the Wnt/β-catenin pathway is the main S100A4-associated pathway. It has also been widely reported that β-catenin in cytoplasm can bind E-cadherin on various epithelial membranes to mediate intercellular adhesion (70, 71). Therefore, we believe that the Wnt pathway regulates EMT from two aspects: on the one hand, the activation of the Wnt pathway leads to the translocation of β-catenin to the nucleus where it regulates the transcription of genes involved in the EMT. On the other hand, the activation of the Wnt pathway promotes the translocation of β-catenin to the nucleus, inhibits the binding of β-catenin to the epithelial cell membrane E-cadherin, weakens the adhesion of epithelial cells, and affects the EMT process. In addition, it has been shown that downregulation of S100A4 can reduce myocardial fibrosis in mice through the Wnt/β-catenin pathway, and knockdown of S100A4 can significantly reduce myocardial fibrosis and β-catenin levels (72). S100A4 interacts with the Wnt/β-catenin pathway to modulate cell migration in cancer and fibrotic diseases (73). The S100A4 gene binds to TCF and β-catenin for transcription (74), but this study was not performed in CRS. We first analyzed the binding of the transcription factor TCF-4 to the S100A4 promoter using the ChIP assay on LPS-stimulated HNEpCs, as well as by Co-IP experiments and found that TCF-4 specifically binds β-catenin from the immunoprecipitates of HNEpCs. The data show that S100A4 is directly regulated by the Wnt/GSK-3β/β-catenin/TCF-4 pathway, which induces mesenchymal transformation of the epithelium into fibroblasts that secrete COL1A1, causing extracellular collagen deposition and tissue remodeling (**Figure 7**).

S100A4 in CRS triggers a series of processes that activate inflammatory responses, such as activation of the Wnt/β-catenin signaling pathway and lead to persistent EMT. This study focuses only on the study of S100A4 in CRS in general and lacks a classification of the different subtypes of CRS. Since S100A4 is an effective inducer of CRS, it would be interesting to find out whether S100A4 is differentially expressed in different immune response types of sinusitis. Then, we can study the interaction between S100A4 and various inflammatory factors in eosinophilic type 2 reactive sinusitis and non-eosinophilic sinusitis. In fact, the increase in S100A4 expression provides an attractive therapeutic target for CRS by neutralizing S100A4 activity, making it an excellent target against tissue remodeling in CRS. S100A4 inhibitors, such as sulinic acid, cloniclosamide and niclosamide, are currently undergoing clinical trials (75, 76). Although the entire study has verified the important role of S100A4 in the occurrence of EMT in CRS at the pathological and cellular levels, the *in vivo* experimental verification is insufficient. Therefore, we next tend to use animals to verify the role of S100A4 in the development of CRS. Specifically, we are able to establish a mouse model of CRS for symptom assessment after anti-S100A4 drug treatment and collecte nasal mucosal biopsies at different time points for proteomic time-series evaluation of the natural variability in nasal mucosal tissue remodeling over time and local response to anti-S100A4 treatment in mice. This also provides clinicians with new ideas for studying CRS, such as focusing on the expression of S100A4 in different CRS disease types, the association between high S100A4 expression and different symptom scores, and the presence of differential S100A4 expression in patients responding to drug treatment.

## Conclusion

s100a4 is involved in the EMT process in CRS mucosa, where it triggers the deposition of COL1A1 in the nasal mucosa and regulates the EMT of HNEpCs *via* the Wnt/β-catenin/TCF-4 signaling pathway.

## Data Availability Statement

The datasets presented in this study can be found in online repositories. The names of the repository/repositories and accession number(s) can be found below: ProteomeXchange; PXD030884 (77–79).

## Ethics Statement

The studies involving human participants were reviewed and approved by the Ethics Committee of Shandong Provincial Hospital Affiliated to Shandong First Medical University. The patients/participants provided their written informed consent to participate in this study.

## Author Contributions

NYG and LS: drafted the important content of the manuscript and explained it, and carried out rigorous conception and design of the subject. XB, HL, and HD: carried out a detailed analysis of the data in the article. PZ and HY: carried out the collection of clinical samples. NG and HH: conducted experimental operations. In addition, MX and CL: provided the subject ideas and careful proofreading of the manuscript. All authors contributed to the article and approved the submitted version.

## Funding

This work was supported by the grants from the Medical Science and Technology Innovation Center, Shandong First Medical University & Shandong Academy of Medical Sciences, the National Natural Science Foundation of China (#81770979, #82071013, #81900922), Natural Science Foundation of Shandong Province (#ZR2019BH019) and Taishan Scholar Foundation of Shandong Province (#tsqn201812134).

## Conflict of Interest

Author HD was employed by the company Qilu Pharmaceutical Co., Ltd.

The remaining authors declare that the research was conducted in the absence of any commercial or financial relationships that could be construed as a potential conflict of interest.

## Publisher’s Note

All claims expressed in this article are solely those of the authors and do not necessarily represent those of their affiliated organizations, or those of the publisher, the editors and the reviewers. Any product that may be evaluated in this article, or claim that may be made by its manufacturer, is not guaranteed or endorsed by the publisher.

## References

[1] Shi JB Fu QL Zhang H Cheng L Wang YJ Zhu DD. Epidemiology of Chronic Rhinosinusitis: Results From a Cross-Sectional Survey in Seven Chinese Cities. Allergy (2015) 70(5):533–9. doi: 10.1111/all.12577 PMC4409092 25631304

[2] Bachert C Marple B Schlosser RJ Hopkins C Schleimer RP Lambrecht BN. Adult Chronic Rhinosinusitis. Nat Rev Dis Primers (2020) 6(1):86. doi: 10.1038/s41572-020-00218-1 33122665

[3] Zhang L Zhang Y Gao Y Wang K Lou H Meng Y. Long-Term Outcomes of Different Endoscopic Sinus Surgery in Recurrent Chronic Rhinosinusitis With Nasal Polyps and Asthma. Rhinology (2020) 58(2):126–35. doi: 10.4193/Rhin19.184 31904028

[4] Jiao J Wang C Zhang L. Epithelial Physical Barrier Defects in Chronic Rhinosinusitis. Expert Rev Clin Immunol (2019) 15(6):679–88. doi: 10.1080/1744666X.2019.1601556 30925220

[5] Hammad H Lambrecht BN. Barrier Epithelial Cells and the Control of Type 2 Immunity. Immunity (2015) 43(1):29–40. doi: 10.1016/j.immuni.2015.07.007 26200011

[6] Holgate ST. The Sentinel Role of the Airway Epithelium in Asthma Pathogenesis. Immunol Rev (2011) 242(1):205–19. doi: 10.1111/j.1600-065X.2011.01030.x 21682747

[7] Grossman J. One Airway, One Disease. Chest (1997) 111(2 Suppl):11S–6S. doi: 10.1378/chest.111.2_supplement.11s 9042022

[8] Fokkens WJ Lund VJ Hopkins C Hellings PW Kern R Toppila-Salmi S. European Position Paper on Rhinosinusitis and Nasal Polyps 2020. Rhinology (2020) 58(Suppl S29):1–464. doi: 10.4193/Rhin20.600 32077450

[9] Kao SS Bassiouni A Ramezanpour M Chegeni N Colella AD Chataway TK. Scoping Review of Chronic Rhinosinusitis Proteomics. Rhinology (2020) 58(5):418–29. doi: 10.4193/Rhin20.034 32500870

[10] Kao SS Bassiouni A Ramezanpour M Finnie J Chegeni N Colella AD. Proteomic Analysis of Nasal Mucus Samples of Healthy Patients and Patients With Chronic Rhinosinusitis. J Allergy Clin Immunol (2021) 147(1):168–78. doi: 10.1016/j.jaci.2020.06.037 32750382

[11] Shigetomi K Ikenouchi J. Regulation of the Epithelial Barrier by Post-Translational Modifications of Tight Junction Membrane Proteins. J Biochem (2018) 163(4):265–72. doi: 10.1093/jb/mvx077 29186552

[12] Niessen CM. Tight Junctions/Adherens Junctions: Basic Structure and Function. J Invest Dermatol (2007) 127(11):2525–32. doi: 10.1038/sj.jid.5700865 17934504

[13] Barmeyer C Schulzke JD Fromm M. Claudin-Related Intestinal Diseases. Semin Cell Dev Biol (2015) 42:30–8. doi: 10.1016/j.semcdb.2015.05.006 25999319

[14] Lamouille S Xu J Derynck R. Molecular Mechanisms of Epithelial-Mesenchymal Transition. Nat Rev Mol Cell Biol (2014) 15(3):178–96. doi: 10.1038/nrm3758 PMC4240281 24556840

[15] Fei F Qu J Zhang M Li Y Zhang S. S100A4 in Cancer Progression and Metastasis: A Systematic Review. Oncotarget (2017) 8(42):73219–39. doi: 10.18632/oncotarget.18016 PMC5641208 29069865

[16] Davies BR Davies MP Gibbs FE Barraclough R Rudland PS. Induction of the Metastatic Phenotype by Transfection of a Benign Rat Mammary Epithelial Cell Line With the Gene for p9Ka, a Rat Calcium-Binding Protein, But Not With the Oncogene EJ-Ras-1. Oncogene (1993) 8(4):999–1008. 8455951

[17] Grigorian M Ambartsumian N Lykkesfeldt AE Bastholm L Elling F Georgiev G. Effect of Mts1 (S100A4) Expression on the Progression of Human Breast Cancer Cells. Int J Cancer (1996) 67(6):831–41. doi: 10.1002/(SICI)1097-0215(19960917)67:6<831::AID-IJC13>3.0.CO;2-4 8824556

[18] Maelandsmo GM Hovig E Skrede M Engebraaten O Flørenes VA Myklebost O. Reversal of the *In Vivo* Metastatic Phenotype of Human Tumor Cells by an Anti-CAPL (Mts1) Ribozyme. Cancer Res (1996) 56(23):5490–8. 8968106

[19] Takenaga K Nakamura Y Endo H Sakiyama S. Involvement of S100-Related Calcium-Binding Protein Pel98 (or Mts1) in Cell Motility and Tumor Cell Invasion. Jpn J Cancer Res (1994) 85(8):831–9. doi: 10.1111/j.1349-7006.1994.tb02955.x PMC5919561 7928629

[20] Bertheloot D Latz E. Hmgb1, Il-1α, IL-33 and S100 Proteins: Dual-Function Alarmins. Cell Mol Immunol (2017) 14(1):43–64. doi: 10.1038/cmi.2016.34 27569562 PMC5214941

[21] Austermann J Zenker S Roth J. S100-Alarmins: Potential Therapeutic Targets for Arthritis. Expert Opin Ther Targets (2017) 21(7):739–51. doi: 10.1080/14728222.2017.1330411 28494625

[22] Gong T Liu L Jiang W Zhou R. DAMP-Sensing Receptors in Sterile Inflammation and Inflammatory Diseases. Nat Rev Immunol (2020) 20(2):95–112. doi: 10.1038/s41577-019-0215-7 31558839

[23] Boteanu RM Suica VI Uyy E Ivan L Dima SO Popescu I. Alarmins in Chronic Noncommunicable Diseases: Atherosclerosis, Diabetes and Cancer. J Proteomics (2017) 153:21–9. doi: 10.1016/j.jprot.2016.11.006 27840210

[24] Raposo TP Beirão BC Pang LY Queiroga FL Argyle DJ. Inflammation and Cancer: Till Death Tears Them Apart. Vet J (2015) 205(2):161–74. doi: 10.1016/j.tvjl.2015.04.015 25981934

[25] Hansen MT Forst B Cremers N Quagliata L Ambartsumian N Grum-Schwensen B. And Metastasis: Serum Amyloid A1 and A3 Induce Metastasis, and are Targets of Metastasis-Inducing S100A4. Oncogene (2015) 34(4):424–35. doi: 10.1038/onc.2013.568 24469032

[26] Sumsion JS Pulsipher A Alt JA. Differential Expression and Role of S100 Proteins in Chronic Rhinosinusitis. Curr Opin Allergy Clin Immunol (2020) 20(1):14–22. doi: 10.1097/ACI.0000000000000595 31644435 PMC7179082

[27] Van Crombruggen K Vogl T Pérez-Novo C Holtappels G Bachert C. Differential Release and Deposition of S100A8/A9 Proteins in Inflamed Upper Airway Tissue. Eur Respir J (2016) 47(1):264–74. doi: 10.1183/13993003.00159-2015 26493794

[28] Dahlmann M Kobelt D Walther W Mudduluru G Stein U. S100A4 in Cancer Metastasis: Wnt Signaling-Driven Interventions for Metastasis Restriction. Cancers (Basel) (2016) 8(6):59. doi: 10.3390/cancers8060059 PMC4931624 27331819

[29] Yeh Y Guo Q Connelly Z Cheng S Yang S Prieto-Dominguez N. Wnt/Beta-Catenin Signaling and Prostate Cancer Therapy Resistance. Adv Exp Med Biol (2019) 1210:351–78. doi: 10.1007/978-3-030-32656-2_16 31900917

[30] Gao C Chen YG. Dishevelled: The Hub of Wnt Signaling. Cell Signal (2010) 22(5):717–27. doi: 10.1016/j.cellsig.2009.11.021 20006983

[31] Wong SHM Fang CM Chuah LH Leong CO Ngai SC. E-Cadherin: Its Dysregulation in Carcinogenesis and Clinical Implications. Crit Rev Oncol Hematol (2018) 121:11–22. doi: 10.1016/j.critrevonc.2017.11.010 29279096

[32] Harada K. Sclerosing and Obstructive Cholangiopathy in Biliary Atresia: Mechanisms and Association With Biliary Innate Immunity. Pediatr Surg Int (2017) 33(12):1243–8. doi: 10.1007/s00383-017-4154-8 29039048

[33] Ning Q Li F Wang L Li H Yao Y Hu T. S100A4 Amplifies TGF-β-Induced Epithelial-Mesenchymal Transition in a Pleural Mesothelial Cell Line. J Investig Med (2018) 66(2):334–9. doi: 10.1136/jim-2017-000542 PMC5800353 29141874

[34] Liu J Shen JX Wu HT Li XL Wen XF Du CW. Collagen 1A1 (COL1A1) Promotes Metastasis of Breast Cancer and is a Potential Therapeutic Target. Discov Med (2018) 25(139):211–23. 29906404

[35] Yang F Xu J Li H Tan M Xiong X Sun Y. FBXW2 Suppresses Migration and Invasion of Lung Cancer Cells *via* Promoting β-Catenin Ubiquitylation and Degradation. Nat Commun (2019) 10(1):1382. doi: 10.1038/s41467-019-09289-5 30918250 PMC6437151

[36] García de Herreros A Duñach M. Intracellular Signals Activated by Canonical Wnt Ligands Independent of GSK3 Inhibition and β-Catenin Stabilization. Cells (2019) 8(10):1148. doi: 10.3390/cells8101148 PMC6829497 31557964

[37] Liu P Liang B Liu M Lebbink JHG Li S Qian M. Oncogenic Mutations in Armadillo Repeats 5 and 6 of β-Catenin Reduce Binding to APC, Increasing Signaling and Transcription of Target Genes. Gastroenterology (2020) 158(4):1029–43. doi: 10.1053/j.gastro.2019.11.302 PMC7179799 31857074

[38] Zhang LN Huang YH Zhao L. Fusion of Macrophages Promotes Breast Cancer Cell Proliferation, Migration and Invasion Through Activating Epithelial-Mesenchymal Transition and Wnt/β-Catenin Signaling Pathway. Arch Biochem Biophys (2019) 676:108137. doi: 10.1016/j.abb.2019.108137 31605677

[39] Niu J Li XM Wang X Liang C Zhang YD Li HY. DKK1 Inhibits Breast Cancer Cell Migration and Invasion Through Suppression of β-Catenin/MMP7 Signaling Pathway. Cancer Cell Int (2019) 19:168. doi: 10.1186/s12935-019-0883-1 31285694 PMC6591985

[40] Zhou M Tan KS Guan WJ Jiang LJ Deng J Gao WX. Proteomics Profiling of Epithelium-Derived Exosomes From Nasal Polyps Revealed Signaling Functions Affecting Cellular Proliferation. Respir Med (2020) 162:105871. doi: 10.1016/j.rmed.2020.105871 32056672

[41] Nieto MA Huang RY Jackson RA Thiery JP. Emt: 2016. Cell (2016) 166(1):21–45. doi: 10.1016/j.cell.2016.06.028 27368099

[42] Jolly MK Ware KE Gilja S Somarelli JA Levine H. EMT and MET: Necessary or Permissive for Metastasis? Mol Oncol (2017) 11(7):755–69. doi: 10.1002/1878-0261.12083 PMC5496498 28548345

[43] Forte E Chimenti I Rosa P Angelini F Pagano F Calogero A. EMT/MET at the Crossroad of Stemness, Regeneration and Oncogenesis: The Ying-Yang Equilibrium Recapitulated in Cell Spheroids. Cancers (Basel) (2017) 9(8):98. doi: 10.3390/cancers9080098 PMC5575601 28758926

[44] Khalmuratova R Park JW Shin HW. Immune Cell Responses and Mucosal Barrier Disruptions in Chronic Rhinosinusitis. Immune Netw (2017) 17(1):60–7. doi: 10.4110/in.2017.17.1.60 PMC5334123 28261021

[45] Le Hir M Hegyi I Cueni-Loffing D Loffing J Kaissling B. Characterization of Renal Interstitial Fibroblast-Specific Protein 1/S100A4-Positive Cells in Healthy and Inflamed Rodent Kidneys. Histochem Cell Biol (2005) 123(4-5):335–46. doi: 10.1007/s00418-005-0788-z 15856273

[46] Semov A Moreno MJ Onichtchenko A Abulrob A Ball M Ekiel I. Metastasis-Associated Protein S100A4 Induces Angiogenesis Through Interaction With Annexin II and Accelerated Plasmin Formation. J Biol Chem (2005) 280(21):20833–41. doi: 10.1074/jbc.M412653200 15788416

[47] Chow KH HJ P George J Yamamoto K Gallup AD Graber JH. S100A4 Is a Biomarker and Regulator of Glioma Stem Cells That Is Critical for Mesenchymal Transition in Glioblastoma. Cancer Res (2017) 77(19):5360–73. doi: 10.1158/0008-5472.CAN-17-1294 PMC5626628 28807938

[48] Donato R Cannon BR Sorci G Riuzzi F Hsu K Weber DJ. Functions of S100 Proteins. Curr Mol Med (2013) 13(1):24–57. doi: 10.2174/156652413804486214 22834835 PMC3707951

[49] Bowers RR Manevich Y Townsend DM Tew KD. Sulfiredoxin Redox-Sensitive Interaction With S100A4 and Non-Muscle Myosin IIA Regulates Cancer Cell Motility. Biochemistry (2012) 51(39):7740–54. doi: 10.1021/bi301006w PMC3472422 22934964

[50] Fei F Qu J Li C Wang X Li Y Zhang S. Role of Metastasis-Induced Protein S100A4 in Human Non-Tumor Pathophysiologies. Cell Biosci (2017) 7:64. doi: 10.1186/s13578-017-0191-1 29204268 PMC5702147

[51] Bresnick AR Weber DJ Zimmer DB. S100 Proteins in Cancer. Nat Rev Cancer (2015) 15(2):96–109. doi: 10.1038/nrc3893 25614008 PMC4369764

[52] Nasser MW Elbaz M Ahirwar DK Ganju RK. Conditioning Solid Tumor Microenvironment Through Inflammatory Chemokines and S100 Family Proteins. Cancer Lett (2015) 365(1):11–22. doi: 10.1016/j.canlet.2015.05.002 25963887 PMC11707611

[53] Kriajevska M Fischer-Larsen M Moertz E Vorm O Tulchinsky E Grigorian M. Liprin Beta 1, a Member of the Family of LAR Transmembrane Tyrosine Phosphatase-Interacting Proteins, Is a New Target for the Metastasis-Associated Protein S100A4 (Mts1) J. Biol Chem (2002) 277:5229–35. doi: 10.1074/jbc.M110976200 11836260

[54] Gong XJ Song XY Wei H Wang J Niu M. Serum S100A4 Levels as a Novel Biomarker for Detection of Acute Myocardial Infarction. Eur Rev Med Pharmacol Sci (2015) 19(12):2221–5. 26166646

[55] Christensen MH Fenne IS Nordbø Y Varhaug JE Nygård KO Lien EA. Novel Inflammatory Biomarkers in Primary Hyperparathyroidism. Eur J Endocrinol (2015) 173(1):9–17. doi: 10.1530/EJE-14-1038 25850829

[56] Zhang J Hou S Gu J Tian T Yuan Q Jia J. S100A4 Promotes Colon Inflammation and Colitis-Associated Colon Tumorigenesis. Oncoimmunology (2018) 7(8):e1461301. doi: 10.1080/2162402X.2018.1461301 30221056 PMC6136879

[57] Yammani RR Long D Loeser RF. Interleukin-7 Stimulates Secretion of S100A4 by Activating the JAK/STAT Signaling Pathway in Human Articular Chondrocytes. Arthritis Rheumatol (2009) 60(3):792–800. doi: 10.1002/art.24295 PMC2676111 19248116

[58] Hoggard M Jacob B Wheeler D Zoing M Chang K Biswas K. Multiomic Analysis Identifies Natural Intrapatient Temporal Variability and Changes in Response to Systemic Corticosteroid Therapy in Chronic Rhinosinusitis. Immun Inflamm Dis (2021) 9(1):90–107. doi: 10.1002/iid3.349 33220024 PMC7860613

[59] Pierce J Fakhari M Works K Pierce J Clancy R. Understanding Proteomics. Nurs. Health Sci (2007) 9:54–60. doi: 10.1111/j.1442-2018.2007.00295.x 17300546

[60] Wang Z Chen Z Li B Zhang B Du Y Liu Y. Curcumin Attenuates Renal Interstitial Fibrosis of Obstructive Nephropathy by Suppressing Epithelial-Mesenchymal Transition Through Inhibition of the TLR4/NF-Кb and PI3K/AKT Signalling Pathways. Pharm Biol (2020) 58(1):828–37. doi: 10.1080/13880209.2020.1809462 PMC7470153 32866059

[61] Xiao K He W Guan W Hou F Yan P Xu J. Mesenchymal Stem Cells Reverse EMT Process Through Blocking the Activation of NF-κb and Hedgehog Pathways in LPS-Induced Acute Lung Injury. Cell Death Dis (2020) 11(10):863. doi: 10.1038/s41419-020-03034-3 33060560 PMC7567061

[62] Grum-Schwensen B Klingelhöfer J Beck M Bonefeld CM Hamerlik P Guldberg P. S100A4-Neutralizing Antibody Suppresses Spontaneous Tumor Progression, Pre-Metastatic Niche Formation and Alters T-Cell Polarization Balance. BMC Cancer (2015) 15:44. doi: 10.1186/s12885-015-1034-2 25884510 PMC4335362

[63] Tomcik M Palumbo-Zerr K Zerr P Avouac J Dees C Sumova B. S100A4 Amplifies TGF-β-Induced Fibroblast Activation in Systemic Sclerosis. Ann Rheum Dis (2015) 74(9):1748–55. doi: 10.1136/annrheumdis-2013-204516 24709861

[64] Wight TN Frevert CW Debley JS Reeves SR Parks WC Ziegler SF. Interplay of Extracellular Matrix and Leukocytes in Lung Inflammation. Cell Immunol (2017) 312:1–14. doi: 10.1016/j.cellimm.2016.12.003 28077237 PMC5290208

[65] Yammani RR Carlson CS Bresnick AR Loeser RF. Increase in Production of Matrix Metalloproteinase 13 by Human Articular Chondrocytes Due to Stimulation With S100A4: Role of the Receptor for Advanced Glycation End Products. Arthritis Rheumatol (2006) 54(9):2901–11. doi: 10.1002/art.22042 16948116

[66] Nissinen L Kähäri VM. Matrix Metalloproteinases in Inflammation. Biochim Biophys Acta (2014) 1840(8):2571–80. doi: 10.1016/j.bbagen.2014.03.007 24631662

[67] Schmidt-Hansen B Klingelhöfer J Grum-Schwensen B Christensen A Andresen S Kruse C. Functional Significance of Metastasis-Inducing S100A4(Mts1) in Tumor-Stroma Interplay. J Biol Chem (2004) 279(23):24498–504. doi: 10.1074/jbc.M400441200 15047714

[68] Schmidt-Hansen B Ornås D Grigorian M Klingelhöfer J Tulchinsky E Lukanidin E. Extracellular S100A4(Mts1) Stimulates Invasive Growth of Mouse Endothelial Cells and Modulates MMP-13 Matrix Metalloproteinase Activity. Oncogene (2004) 23(32):5487–95. doi: 10.1038/sj.onc.1207720 15122322

[69] Carroll WW O’Connell BP Schlosser RJ Gudis DA Karnezis TT Lawrence LA. Fibroblast Levels are Increased in Chronic Rhinosinusitis With Nasal Polyps and Are Associated With Worse Subjective Disease Severity. Int Forum Allergy Rhinol (2016) 6(2):162–8. doi: 10.1002/alr.21636 26370180

[70] Zhao Y Yu T Zhang N Chen J Zhang P Li S. Nuclear E-Cadherin Acetylation Promotes Colorectal Tumorigenesis *via* Enhancing β-Catenin Activity. Mol Cancer Res (2019) 17(2):655–65. doi: 10.1158/1541-7786.MCR-18-0637 30401720

[71] Kim WK Kwon Y Jang M Park M Kim J Cho S. β-Catenin Activation Down-Regulates Cell-Cell Junction-Related Genes and Induces Epithelial-to-Mesenchymal Transition in Colorectal Cancers. Sci Rep (2019) 9(1):18440. doi: 10.1038/s41598-019-54890-9 31804558 PMC6895046

[72] Qian L Hong J Zhang Y Zhu M Wang X Zhang Y. Downregulation of S100A4 Alleviates Cardiac Fibrosis *via* Wnt/β -Catenin Pathway in Mice. Cell Physiol Biochem (2018) 46(6):2551–60. doi: 10.1159/000489683 29758552

[73] Sack U Walther W Scudiero D Selby M Aumann J Lemos C. S100A4-Induced Cell Motility and Metastasis Is Restricted by the Wnt/β-Catenin Pathway Inhibitor Calcimycin in Colon Cancer Cells. Mol Biol Cell (2011) 22(18):3344–54. doi: 10.1091/mbc.E10-09-0739 PMC3172260 21795396

[74] Stein U Arlt F Walther W Smith J Waldman T Harris ED. The Metastasis-Associated Gene S100A4 Is a Novel Target of Beta-Catenin/T-Cell Factor Signaling in Colon Cancer. Gastroenterology (2006) 131:1486–500. doi: 10.1053/j.gastro.2006.08.041 17101323

[75] Burock S Daum S Keilholz U Neumann K Walther W Stein U. Phase II Trial to Investigate the Safety and Efficacy of Orally Applied Niclosamide in Patients With Metachronous or Sychronous Metastases of a Colorectal Cancer Progressing After Therapy: The NIKOLO Trial. BMC Cancer (2018) 18(1):297. doi: 10.1186/s12885-018-4197-9 29544454 PMC5856000

[76] Stewart RL Carpenter BL West DS Knifley T Liu L Wang C. S100A4 Drives Non-Small Cell Lung Cancer Invasion, Associates With Poor Prognosis, and Is Effectively Targeted by the FDA-Approved Anti-Helminthic Agent Niclosamide. Oncotarget (2016) 7(23):34630–42. doi: 10.18632/oncotarget.8969 PMC5085181 27127879

[77] Homsi MT Gaffey MM. Sinus Endoscopic Surgery. In: Statpearls [Internet]. Treasure Island (FL: StatPearls Publishing (2020). 33085349

[78] Zhu Y Qi X Yu C Yu S Zhang C Zhang Y. Identification of Prothymosin Alpha (PTMA) as a Biomarker for Esophageal Squamous Cell Carcinoma (ESCC) by Label-Free Quantitative Proteomics and Quantitative Dot Blot (QDB). Clin Proteomics (2019) 16:12. doi: 10.1186/s12014-019-9232-6 30988666 PMC6449931

[79] Deutsch EW Bandeira N Sharma V Perez-Riverol Y Carver JJ Kundu DJ. The Proteomexchange Consortium in 2020: Enabling ‘Big Data’ Approaches in Proteomics. Nucleic Acids Res (2020) 48(D1):D1145–52. doi: 10.1093/nar/gkz984 PMC7145525 31686107

